# The effect of thiamine deficiency on inflammation, oxidative stress and cellular migration in an experimental model of sepsis

**DOI:** 10.1186/1476-9255-11-11

**Published:** 2014-04-24

**Authors:** José Antenor Araújo de Andrade, Carlos Roberto Machado Gayer, Natália Pereira de Almeida Nogueira, Márcia Cristina Paes, Vera Lúcia Freire Cunha Bastos, Jayme da Cunha Bastos Neto, Sílvio Caetano Alves, Raphael Molinaro Coelho, Mariana Gysele Amarante Teixeira da Cunha, Rachel Novaes Gomes, Márcia Barbosa Águila, Carlos Alberto Mandarim-de-Lacerda, Patrícia Torres Bozza, Sérgio da Cunha

**Affiliations:** 1 Intensive Care Unit, Pedro Ernesto University Hospital, State University of Rio de Janeiro, Av. 28 de Setembro 87, Rio de Janeiro, RJ CEP 20551-030, Brazil; 2 Biochemistry Department, Biomedical Center, Institute of Biology, State University of Rio de Janeiro, Rio de Janeiro, Brazil; 3 Immunopharmacology Laboratory, Oswaldo Cruz Institute, Rio de Janeiro, Brazil; 4 Morphometry, Metabolism & Cardiovascular Laboratory; Biomedical Center, Institute of Biology, State University of Rio de Janeiro, Rio de Janeiro, Brazil

**Keywords:** Thiamine, Sepsis, Oxidative stress, Inflammation, Sepsis model

## Abstract

**Background:**

Sepsis is a prevalent condition in critically ill patients and may be associated with thiamine deficiency (TD). The aim of this study was to evaluate the effect of TD on inflammation, oxidative stress and cellular recruitment in a sepsis model.

**Methods:**

The experimental sepsis model, cecal ligation and puncture (CLP), was utilized on mice in comparison with a sham procedure. The following four groups were compared against each other: SHAM with AIN93G complete chow, SHAM with thiamine deficient (TD) chow, CLP with AIN93G complete chow, and CLP with TD chow. Thiamine pyrophosphate (TPP) blood concentrations were determined, and blood and peritoneal fluid were evaluated for differences in TNF-alpha, IL-1, IL-6, KC and MCP-1/CCL2 levels. In addition, the levels of 4-HNE adducts in liver proteins were evaluated by Western Blot.

**Results:**

The mean TPP blood concentration from the mice fed with the complete chow was 303.3 ± 42.6 nmol/L, and TD occurred within 10 days. TNF-α and MCP-1 concentrations in the peritoneal fluid were significantly greater in the CLP with TD chow group when compared with the other groups. The blood IL-1β level, however, was lower in the CLP with TD chow group. Liver 4-HNE levels were highest in the TD chow groups. Blood mononuclear cell numbers, as well as peritoneal total leukocyte, mononuclear cell and neutrophil numbers were greater in the CLP with TD chow group. Peritoneal bacterial colony forming units (CFU) were significantly lower in the CLP with TD chow group.

**Conclusion:**

TD was associated with greater bacterial clearance, oxidative stress and inflammatory response changes.

## Background

Several clinical conditions, such as trauma, burn, severe acute illness and major postoperative surgery generate a series of organic response reactions in response to these injuries, called the systemic inflammatory response syndrome (SIRS). When SIRS is associated with an infection, it is defined as sepsis [1-3]. Epidemiological studies worldwide [4-6] and in Brazil [7,8] indicate that sepsis is a prevalent condition with a high mortality rate that is associated with the emergence of multidrug-resistant pathogens [9,10] while also generating high social and financial costs [11]. Thiamine (vitamin B1) is a water soluble vitamin that does not accumulate in the body; therefore, it has to be ingested daily [12]. Thiamine pyrophosphate (TPP; the biologically active form of vitamin B1) is an important mitochondrial enzyme cofactor [12] with a key role in energy balance. In addition, TPP participates as a transketolase cofactor in the pentose phosphate pathway, a pathway that replenishes NADPH, which is necessary for the recovery of the reduced form of glutathione (GSH) from its oxidized form. TPP is also a cofactor of the α-ketoglutarate dehydrogenase complex [12], theoretically participating in the recovery of cellular reducing power [13].

The prevalence of TD in critically ill patients has been described [14-16] and was associated with increased morbidity and mortality [16]. Recent studies have demonstrated that additional attention is needed in the identification of thiamine deficiency (TD) and that thiamine supplementation is necessary not only in intensive care unit patients but also in patients with heart failure [15,17,18].

Evidence that TD may cause heart failure has been demonstrated [12,19,20], whereby an increase in oxidative stress due to TD led to the apoptosis of cardiomyocytes [21] and produced cardiac remodeling [20,22]. Structural remodeling and the destruction of cardiomyocyte mitochondria were also observed with TD [20]. A similar finding was observed in a study with human neuroblastoma cells cultured under TD conditions [23].

Several neurological disorders have been associated with TD, including acute confusion and changes in eye movement and gait (Wernicke’s encephalopathy). These findings may be associated with chronic memory impairment (Korsakoff’s syndrome) [12,24,25]. Thiamine deficiency may also be associated with brain degenerative conditions such as Parkinson’s disease and Alzheimer’s disease [24-27]. Thiamine deficiency is also associated with lesions of the endoplasmic reticulum of neurons [28]. The influence of TPP in an experimental model of sepsis in dogs has recently been performed [29], and the following outcomes were analyzed: blood pH, oxygen consumption, base excess, mean arterial pressure and the cardiac index. The authors observed recovery of all of these parameters in the animals treated with TPP, suggesting the involvement of thiamine as a possible protective agent against the onset of sepsis.

Sepsis is associated with an intense inflammatory response [30]. In response to an injury, the initial inflammatory response is the activation of the innate immunity pathways. The innate inflammatory response is a series of “primitive” non-specific reactions that occur in the early hours after the interaction with a pathogen and involve acute phase proteins, the complement system, dendritic cells, macrophages and natural killer cells (NK), with the end result being inflammation [31-34]. When an activated macrophage comes into contact with a pathogen, it begins to produce TNF-α, which is responsible for an increase in vascular permeability. In addition, pathogen stimulation of macrophages leads to the production of IL-1β and IL-6, which have decisive roles in the genesis of fever by acting on the central hypothalamic temperature control system [34]. The chemotactic factors KC and MCP-1 are involved in the recruitment of neutrophils and monocytes, respectively, and are involved in the perpetuation of the cellular response to injury. In parallel, IL-10 secretion is a self-regulatory mechanism of the inflammatory response, acting as an anti-inflammatory cytokine [30,31].

The cellular injury observed in sepsis produces highly reactive biochemical intermediates from nitrogen and oxygen [35]. Following an injury, we now know that the increased production of reactive oxygen species (ROS) and the increased activity of clearance mechanisms do not necessarily indicate cell damage [35,36]. ROS have the ability to react with proteins, lipids, and genetic material. The products of these reactions are known today and include 4-hydroxy 2-nonenal (4-HNE), an aldehyde that results from the lipid peroxidation of the cell membrane [37]. 4-HNE plays a key role in cell signaling and redox balance [38,39], but in high concentrations, it can be harmful to cells [37-39].

Experimental sepsis models are necessary to evaluate some aspects of this clinical condition, due to ethical constraints [40-42]. Many methods are used to simulate hemodynamic, immunological and clinical sepsis events, including surgical manipulation of organs (such as ligation or perforation of the bowel loops) [42] and injection of bacteria or their components (e.g., endotoxins, such as gram-negative lipopolysaccharides) into the bloodstream [41], organs and anatomical cavities. These methods are not free of criticisms from both a methodological standpoint [43,44] and from technical and ethical standpoints, with regard to animal handling [45]. These experimental models, however, represent important tools for sepsis investigation.

This study analyzed the possible effect of thiamine deficiency on changes in sepsis markers of inflammation (cytokines), the balance of the oxidation-reduction system (based on the presence of a marker of lipid peroxidation), and the leukocyte recruitment profile. The hypothesis in question was that thiamine deficiency in experimental sepsis conditions could result in a pro-inflammatory profile, with greater oxidative stress and cellular migration.

## Methods

This study was approved by the ethics committee for the use of animals for research at the Roberto Alcântara Gomes Biology Institute – UERJ (CEUA/036/2011).

### Animals and chow

Sixty-five male C57bl6 mice (Cecal – FIOCRUZ, Rio de Janeiro, RJ, Brazil), between 6 and 8 weeks old, were maintained in groups of 5 per cage, with free access to water and food. The mice were exposed to a 12-hour light cycle (lights off until 6 a.m. and a controlled temperature (24°C ± 2°C). Two types of chow were used for the study (Pragsoluções Biociências, Jaú, SP, Brazil). The first type of chow contained all of the components of a normal AIN93G chow [46]. The second chow type was similar to the AIN93G chow but without the addition of thiamine. Before each experiment, all groups were fed with the AIN93G chow for one week to standardize the thiamine intake. Throughout the study, the animal’s body weight and chow intake were measured.

### Determining the time required for TD

To determine the time required to produce a TD, mice were divided into five groups (five animals per group). Initially, all groups were fed with the AIN93G chow for seven days to standardize the thiamine intake. The control group was sacrificed after seven days, and blood was collected for determination of the circulating thiamine levels by High Performance Liquid Chromatography (HPLC). The other four groups were fed with the thiamine deficient AIN93G chow for an additional 10, 15, 20 and 25 days before euthanasia, and blood samples were collected for HPLC analyses. We determined the normal concentration of TPP in C57bl6 mice, which is information that was not found in prior studies. Thiamine deficiency was defined as a TPP blood concentration lower than two standard deviations below the mean.

### The experimental sepsis procedure

For the experimental sepsis model experiments, forty mice were divided into 4 groups (10 animals per group), and the duration of the each experiment was 15 days. The experimental groups were as follows: 1) a control group fed with the AIN93G complete chow that was subjected to laparotomy without cecal ligation and puncture - CLP (sham); 2) a group fed with the AIN93G complete chow that was subjected to laparotomy and CLP; 3) a group fed with the thiamine deficient AIN93G chow that was subjected to laparotomy without CLP; and 4) a group fed with the thiamine deficient AIN93G chow and subjected to laparotomy and CLP. For all groups, euthanasia was performed 24 hours after surgery to collect liver and blood samples.

The animals were anesthetized by intraperitoneal injection of ketamine (65 mg/kg) and xylazine (13 mg/kg) (Vetbrands Brazil, Campinas, SP, Brazil). A cutaneous abdominal incision was made longitudinally and medially, approximately 2 centimeters in length, followed by opening of the peritoneum and cecum identification. The cecum was then ligated to form a closed intestinal loop and was perforated two times by an 18 gauge needle. The closed loop was gently compressed to expose the abdominal cavity to feces, and then the intestinal loop was placed back into the abdominal cavity. The peritoneum and skin were then sutured closed. Sham-operated mice were subjected to identical procedures, except the cecum ligation and puncture steps were omitted. After surgery, a sterile saline subcutaneous injection was given for fluid replacement [42,47]. Euthanasia was performed by an injection of double the amount of anesthetic that was calculated for each animal during the CLP or sham surgeries. Following euthanasia, a blood sample was collected and the abdomen was opened for liver removal.

### Blood sample preparation for HPLC

The blood samples were preserved by refrigeration and processed on the same day of collection. Whole blood was frozen three times in liquid nitrogen to complete hemolysis and was then centrifuged at 14,000 rcf. Then, after thirty minutes of rest, the liquid phase was collected and deproteinated by the addition of 40% trichloroacetic acid (TCA). The TCA was then extracted with five volumes of water-saturated ethylic ether. To form the thiamine thiochrome derivatives, the lower watery fraction was collected and derivatized by adding 50 μL of potassium ferricyanide (30.4 mmol/L) and 50 μL of sodium hydroxide (0.8 mol/L) for each 1,000 μL aliquot of blood. The final sample was frozen at −70°C for later analysis by HPLC [20].

### The determination of TPP by HPLC/fluorescence

After derivatization, 100 μL of the final sample was injected into a Hypersil Gold Amino column (Thermo, USA) (250 × 4.6 mm, 5 μm particle). The shimadzu HPLC system (Shimadzu, Kyoto, Japan) consisted of a LC-10 AD pump valve 535, and a fluorimetric RF-detection method was utilized, based on the excitability of the thiochrome to fluorescent light.

The mobile phase (isocratic), with 30% acetonitrile in a potassium phosphate buffer (0.1 M, pH 7.5), was pumped at a flow rate of 1.0 mL/min. The fluorimetric detection occurred at 450 nm emission and 370 nm excitation wavelengths [20,48]. Standards equivalent to 4.6875, 9.375, 18.75, 37.5, 75, 150, 300 and 600 nmol/L were prepared for the construction of calibration curves.

### The assessment of 4-HNE in the liver by western blot

About 100 mg of hepatic tissue were homogenized in 500 μl of lysis buffer (50 mM HEPES, 1 mM MgCl_2_, 1% Triton X-100, 0.1% SDS, 10 mM EDTA; pH 6.4), containing protease inhibitor cocktail Sigma-Adrich Fine Chemicals (1.04 mM AEBSF, 800 nM aprotinin, 20 μM leupeptin, 40 μM bestatin, 15 μM pepstatin A and 14 μM E-64) in ice. Tissue homogenate was centrifuged at 10,000 rpm for 10 min. the pellet was discarded and the supernatants were used for protein quantification by the Peterson’s method. Whole protein extracts (about 80 μg) were subjected to electrophoresis in a 15% SDS-polyacrylamide gel under reducing conditions. Proteins were transferred (25 mM Tris, 192 mM glicine, pH 8.3, 20% methanol) to a nitrocellulose membrane at 4°C for 2 h. Membranes were blocked with Tris-buffered saline solution containing 0.1% Tween 20 (TBS-T) and 5% bovine serum albumin (BSA). The membranes were then incubated overnight with anti-4-HNE antibody (1:1000), diluted in blocking solution, washed in TBS-T, and finally incubated for 1 h with horseradish peroxidase-conjugated anti-mouse antibody (1:10.000). The bands were revealed by chemiluminescence using the ECL substrate. Blots were exposed to ECL Hyperfilm (Amersham) and quantification was performed by densitometric analysis of the exposed films (Adobe Photoshop 5 programme) and the Ponceau S stained membranes were used as a load control [49].

### Blood and peritoneal fluid cell counts

Blood and peritoneal fluid samples were stained with Giemsa stain for total and differential leukocyte counts by optical microscopy at 1,000x magnification in a Neubauer chamber [47].

### Blood and peritoneal fluid cytokine determination

Determination of the blood and peritoneal fluid TNF-α, IL-1, MCP-1/CCL2, IL-6 and KC levels were performed by ELISA (Duo Set Kit - R & D systems, USA). ELISA plate reading was performed on an ELISA plate reader at 450 nm. The data were analyzed using the Soft Max Pro program, and cytokine concentrations were determined based on their respective standard curves [47].

### Peritoneal fluid colony-forming unit counts

The peritoneal fluid sample colony-forming unit (CFU) counts were obtained by diluting the peritoneal fluid samples 1:50 in sterile PBS, and then the diluted samples were seeded in Petri dishes with TSA growth medium. The plates were incubated at 37°C for 24 hours, after which the number of CFUs was counted. The whole procedure was performed under sterile conditions [47].

### Statistics analyses

A two-way ANOVA with a Sidak post-test was used to analyze the differences between the groups for the biochemical, immunological and cellular assays, to perform a multiple comparison analysis between the variables and to evaluate the contribution of each variable. The software used was the Windows version of Prism 6.00 (GraphPad Software, San Diego, CA, USA). The significance level was set at 0.05 [50].

## Results

### The body weight evolution and chow intake

**Figure 1:**
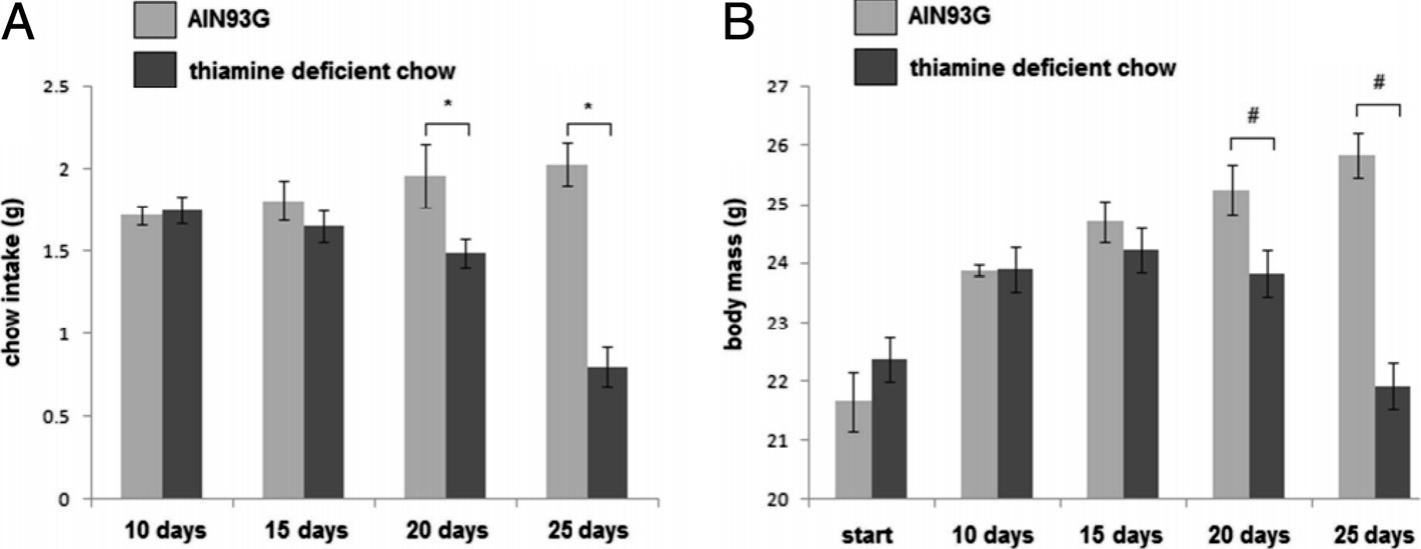
**Evolution of food intake and body mass. (A)** The food intake was similar between groups in 10 and 15 days. Each bar represents five animals (* p < 0.05). **(B)** Body mass gain was similar between groups in 10 and 15 days, and increased in AIN93G group. Each bar represents five animals (# *P* <0.05). Therefore, until the 15th day the animals had a similar nutritional profile. All data are shown as mean ± SEM.

The AIN93G chow intake by the mice increased during the experiments; however, the average TD chow intake was reduced significantly by the 20th and 25th days (Figure 1A). The body mass gain of the animals fed with the AIN93G complete chow increased during the first experiment. Body weight loss occurred at the 20th and 25th days in the animals fed with the TD chow (Figure 1B).

### Determining the efficiency and time required for TD

Initially, the time required for TD was determined. Using HPLC, the mean retention times were 3.4 minutes for free thiamine, 5.3 minutes for thiamine monophosphate, and 6.2 minutes for the TPP. The TPP detection limit was 4.6875 nmol/L. The mean blood TPP concentration in the mice fed the AIN93G complete chow was 303.3 ± 42.6 nmol/L. Thiamine deficiency was observed at 10 days in the mice fed the TD chow, and the blood concentration of TPP in this group at this time point was 12.5 ± 2.4 nmol/L (Table 1).

**Table 1:** Thiamine pyrophosphate whole blood mean concentration

	**TPP nmol/L (mean ± SEM)**	***p *** **(compared to control)**
**Control (AIN93G)**	303.3 ± 19.06	-
**10 days of TD chow**	11.93 ± 1.11	< 0.001
**15 days of TD chow**	5.01 ± 0.67	< 0.001

After 10 days of TD chow, the thiamine pyrophosphate whole blood mean concentration was lower than two standard deviations below the mean, what characterizes thiamine deficiency in these animals.

The TPP blood concentrations were also assessed in the sepsis experiments for validation purposes. As shown in Table 2, both the sham and CLP groups that were fed with TD chow for 15 days showed significantly lower blood TPP concentrations than the sham and CLP groups that were fed with complete chow (Table 2).

**Table 2:** Thiamine pyrophosphate whole blood mean concentration for all groups in sepsis experiment

**Group**	**TPP nmol/L (mean ± SEM)**	***p***
**(1) sham + AIN93G**	383.6 ± 15.39	-
**(2) sham + TD chow**	41.4 ± 10.73	< 0.001 (compared to group 1)
**(3) CPL + AIN93G**	376.9 ± 12.17	-
**(4) CLP + TD chow**	40 ± 6.87	< 0.001 (compared to group 3)

The groups that were fed with TD chow for 15 days showed significantly lower blood TPP concentrations than groups that were fed with complete chow, profile that was similar to that found in the first experiment.

### Effect of TD on 4-HNE-protein adduct formation

**Figure 2:**
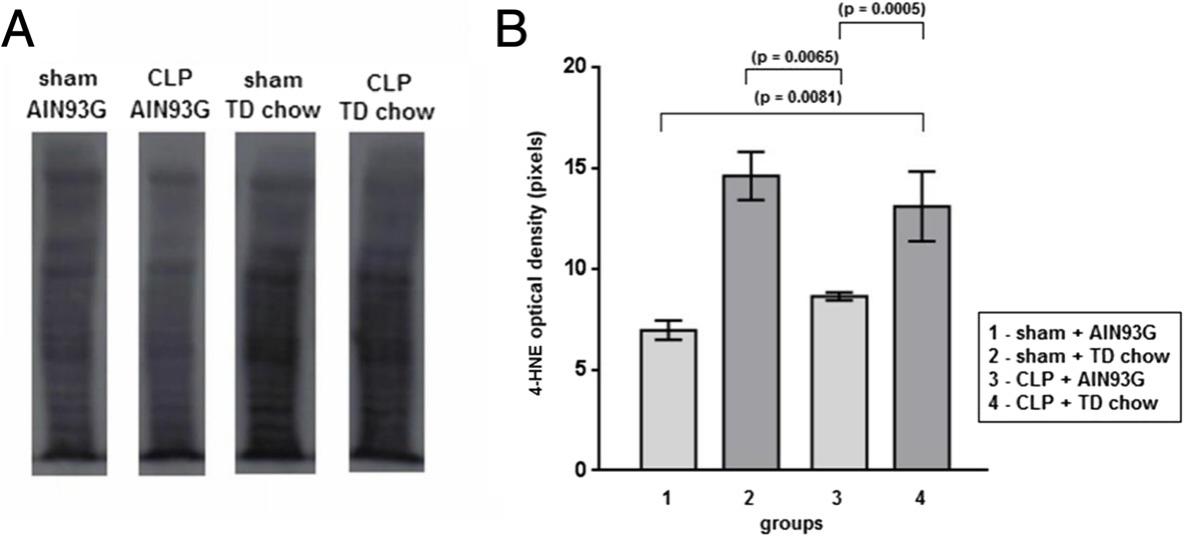
**Determination of 4-HNE binding protein in the liver. (A)** Western blot detail for each group (the image was sliced). **(B)** Optical density analysis for each group, showing a significant increase of 4-HNE in TD chow groups without significant difference between the sham and CLP groups. All data **(B)** are shown as mean ± SEM.

As shown in Figure 2, we observed an increased level of 4-HNE adducts in liver proteins of TD animals when compared with the animals fed with the AIN93G complete chow. There was no significant difference between the sham and CLP groups (Figure 2).

### Blood and peritoneal fluid cell counts

**Figure 3:**
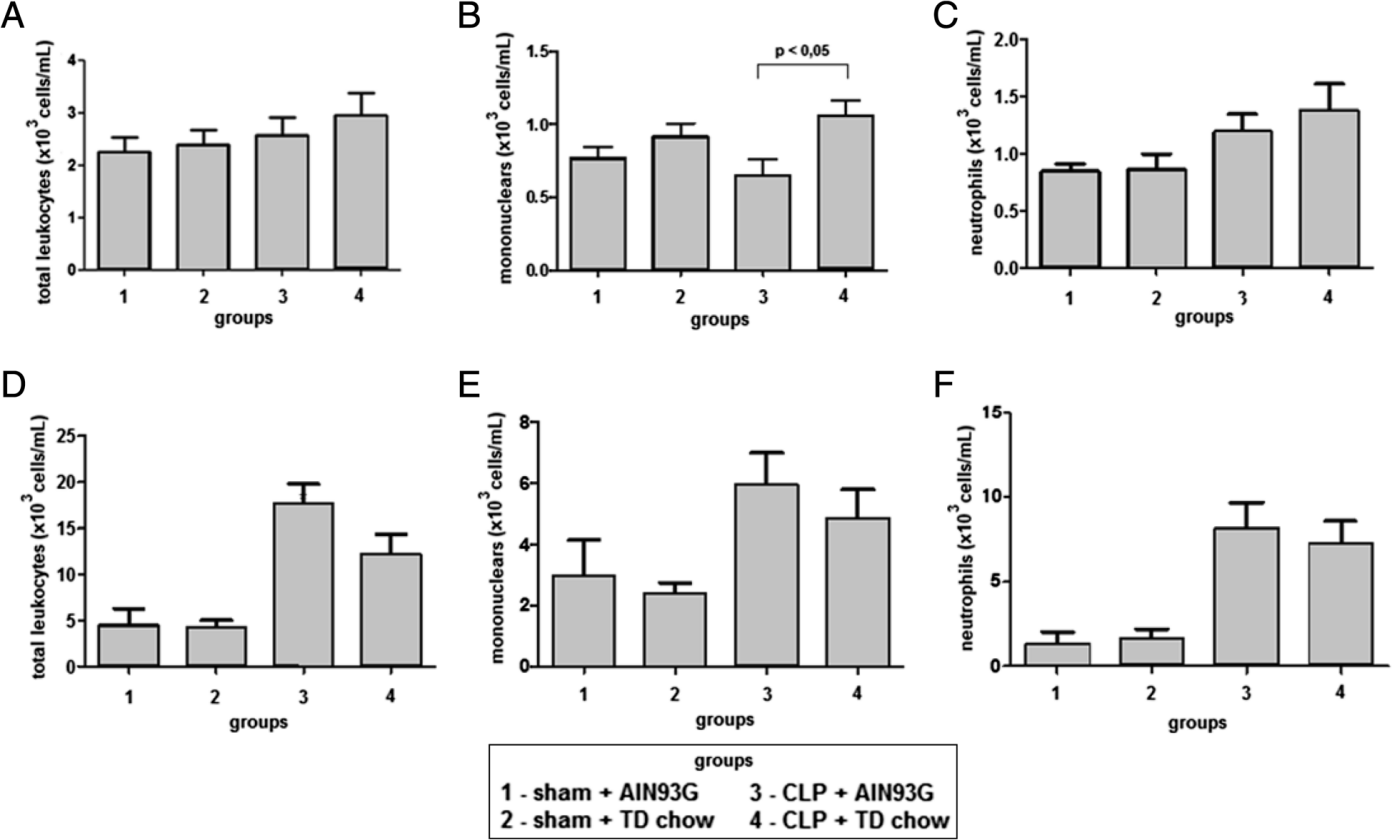
**Total number of leukocytes, mononuclears and neutrophils in peripheral blood (A,B,C) and peritoneal fluid (D,E,F).** Mononuclear cell number in the peripheral blood **(B)** was greater in the CLP group which was fed with the TD chow (*p* < 0,05) but the others cellular types were similar among groups **(A - B)**. In the peritoneal fluid all cellular types were greater in the CLP groups, independent of chow type.

The total number of leukocytes and neutrophils in the peripheral blood was similar in all groups. However, the mononuclear cell number in the peripheral blood was greater in the CLP group that was fed with the TD chow (Figure 3 ABC). In the peritoneal fluid, total leukocyte, mononuclear cell and neutrophil counts were greater in the CLP groups, independent of whether the mice were fed the AIN93G complete or the TD chow (Figure 3 DEF).

### Effect of TD on the blood and peritoneal fluid cytokine levels during sepsis

**Figure 4:**
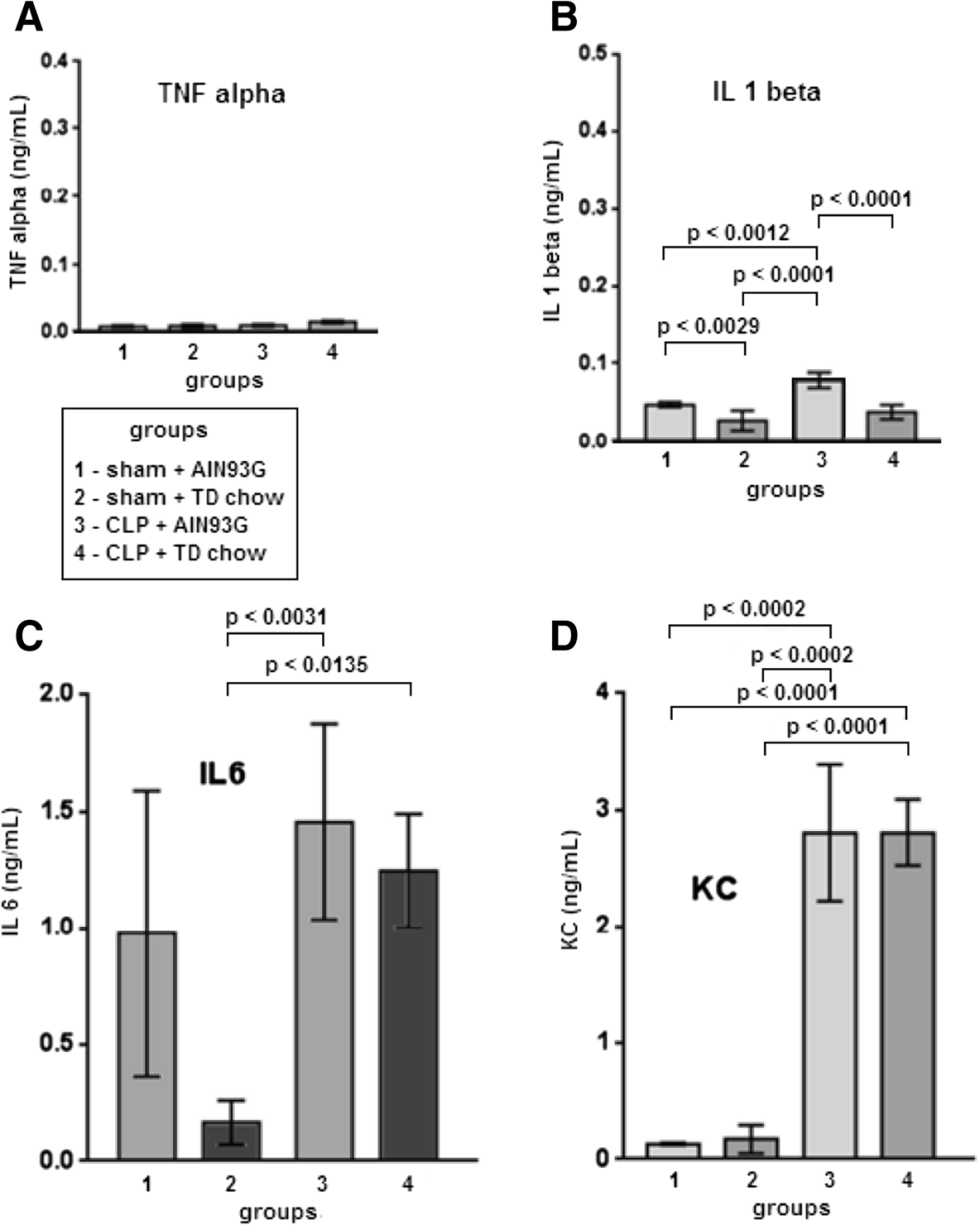
**Blood cytokines 24 h after CLP.** There were no differences in the TNF-α **(A)** blood concentrations in all groups (n = 10 per group). IL-1beta blood concentrations **(B)** were greater in AIN93G groups (independent if sham or CLP) (n = 10 per group). IL-6 blood levels **(C)** tended to be higher in CLP groups (n = 10 per group). KC blood levels **(D)** were higher in CLP groups (n = 5 per group). All data are shown as mean ± SEM.

As shown in Figure 4, 24 h after CLP, the levels of TNF-α, KC, IL-1 β and IL-6 were significantly increased when compared with the sham groups. There were no differences in the TNF-α, KC and IL-6 blood concentrations when the CLP with TD chow group was compared with the CLP with AIN93G complete chow group (Figure 4). Interestingly, the IL-1β blood concentration was greater in the groups that were fed the AIN93G complete chow (independent of sham or CLP surgeries) when compared with the TD chow groups (p < 0.0001) (Figure 4).

**Figure 5:**
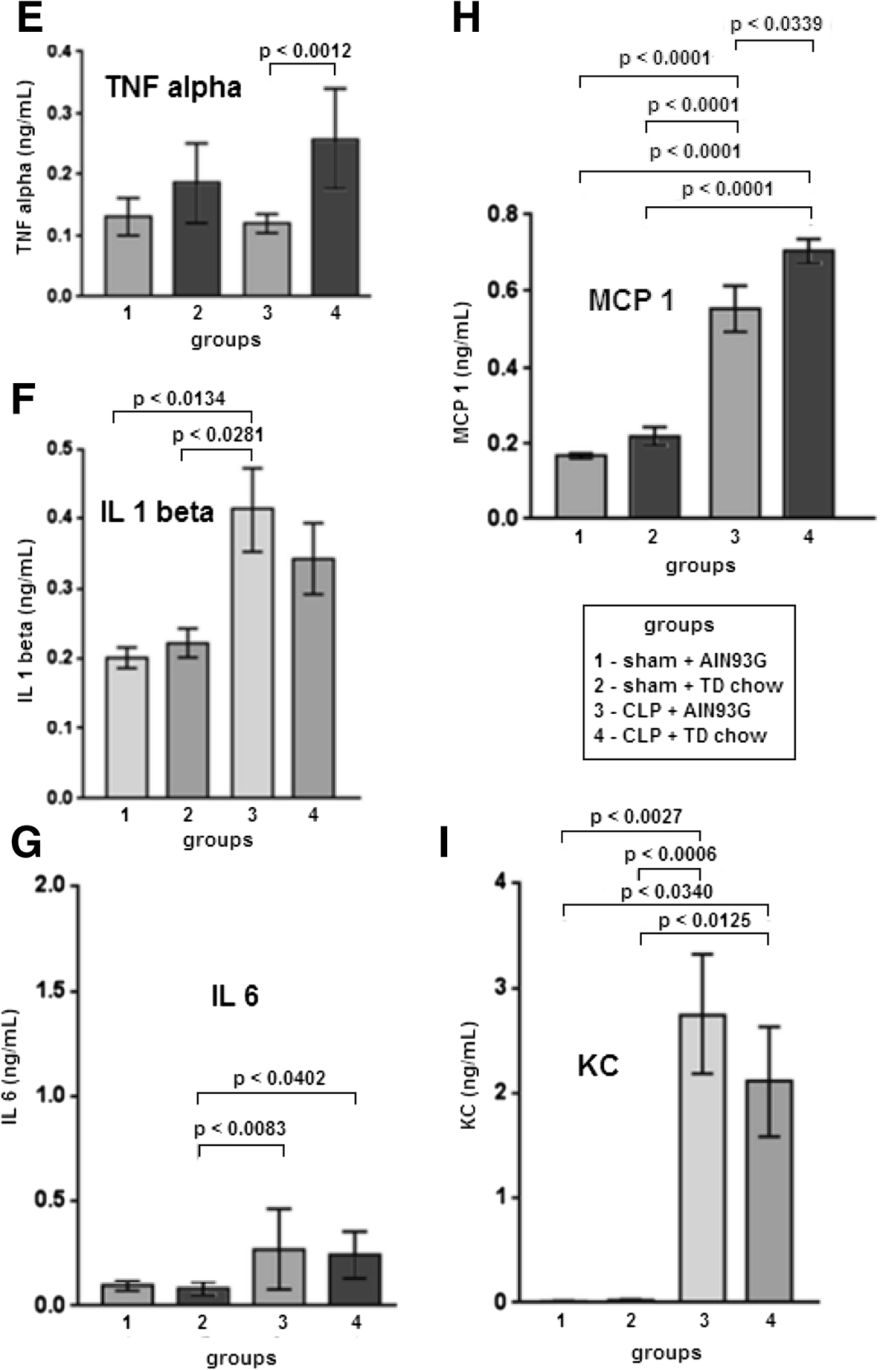
**Peritoneal cytokines 24 h after CLP. (E)** TNF-α peritoneal concentrations were higher in septic animals that were fed with TD chow compared with CLP + AIN93G group (n = 10 per group). IL-1beta **(F)** (n = 10 per group), IL-6 **(G)** (n = 10 per group) and KC **(I)** (n = 5 per group) peritoneal concentrations were higher in CLP groups. MCP-1 **(H)** (n = 5 per group) peritoneal concentrations were higher in septic animals that were fed with TD chow compared with all others groups. All data are shown as mean ± SEM.

In Figure 5, the local peritoneal production of the cytokines was evaluated. The peritoneal concentrations of KC, IL-1β and IL-6 were greater in the CLP groups, independent of whether the mice were fed the complete or TD chow (Figure 5). Interestingly, the MCP-1 and TNF-α peritoneal fluid concentrations were significantly greater in the CLP groups than in the other groups. Both CLP and TD contributed to the elevation of MCP-1 and TNF-α in the peritoneal fluid (Figure 5).

### Peritoneal fluid colony forming unit counts

**Figure 6:**
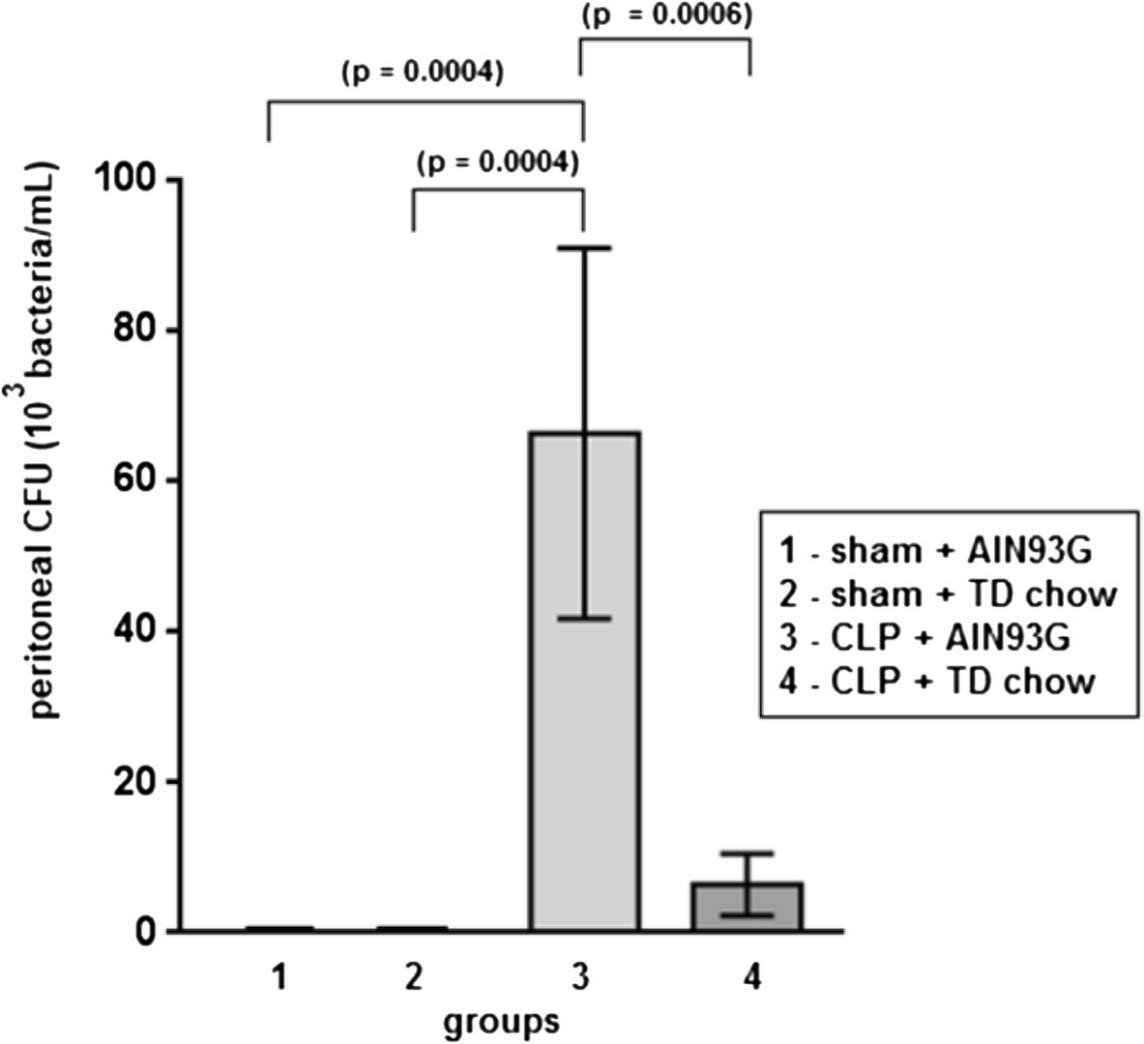
**Peritoneal fluid colony forming unit (CFU) counts.** The septic group that was fed with TD chow showed a lower bacterial concentration in peritoneal fluid, similar to non-septic groups (sham), but the CLP + AIN93G group showed a higher bacterial concentration in peritoneal fluid, what suggests a higher bacterial clearance capacity of the TD chow group. All data are shown as mean ± SEM.

The CLP group with AIN93G complete chow displayed a higher bacterial clearance capacity when compared with all of the other groups, including the CLP group fed the TD chow, where the bacterial clearance was equivalent to the sham animal groups (Figure 6).

## Discussion

In this study, we found that mice fed the thiamine deficient AIN93G chow had significantly reduced levels of the biologically active form of thiamine in their serum, providing an experimental condition to assess the effect of thiamine deficiency during sepsis.

First, the normal blood TPP concentration and the number of days necessary for TD induction with TD chow in the mice were determined. Of note, this information had not yet been described in previous research.

The chow intake and mass gain were progressive in the AIN93G complete chow fed animals. In contrast, a decrease in the chow intake and a loss of body weight throughout the study were observed in the mice fed the thiamine deficient chow. This was a profile similar to what was previously described with Wistar rats [20].

Although the animals fed with the thiamine deficient chow for 10 days displayed a significant reduction of TPP in the blood, the decision to carry out the sepsis experiments after 15 days was made to leave no doubt that thiamine deficiency induction had occurred. Because there was no significant difference in the body mass gain and chow intake between the groups on the 15th day, it was not necessary to perform a paired feeding to avoid errors related to ingestion of different amounts of calories, proteins and other nutrients among the groups.

The levels of IL-6, MCP-1 and KC in the blood and of IL-1β, IL-6 and KC in the peritoneum were not different among the septic groups, independent of their thiamine status. In addition, CLP increased the number of bacteria in the peritoneum, except in the TD group, where the bacterial recovery was similar to that observed in the sham groups.

The level of TNF-α in the peritoneal fluid of the septic animals fed with TD chow was significantly greater than that observed in the other groups. TNF-α is a cytokine of the early innate inflammatory response. It is produced within the first hour of injury, with a peak between the second and third hours following injury. For this reason, it is not usually used in the monitoring of septic patients [51] because the timing of the peak TNF-α level may have already passed when these patients are first evaluated. However, TNF-α is an early marker of inflammation commonly used in sepsis models [47]. TNF-α is a marker of the activity of NK cells and macrophages and may help explain the increased bacterial clearance in the peritoneal fluid that was observed in the CLP mice fed the TD chow used in this study. In addition, we observed a persistent high level of TNF-α in the peritoneal fluid of the CLP mice fed the TD chow, although the number of total leukocytes, monocytes and neutrophils in the peritoneal fluid was similar between the septic animals, independent of their thiamine status. This finding suggests that TD may not change cellular recruitment into the peritoneal cavity following sepsis and that another mechanism may be involved in the increased peritoneal bacterial killing observed in the CLP group fed with TD chow, possibly mediated by TNF-α.

Lipid peroxidation is one of the hallmarks of the oxidative stress. Lipid peroxides are usually decomposed into reactive aldehydes such as malondialdehyde (MDA) and 4-hydroxy-2-nonenal (4-HNE), which are also reactive oxygen species. Due to its electrophilic properties, the aldehyde 4-HNE forms adducts with cellular proteins [52]. The present study also demonstrated a greater level of liver proteins modified by 4-HNE in the animals subjected to TD chow compared with what was observed in the other groups. This finding was expected, considering that thiamine may be associated with a lower efficiency of cellular reducing power, since enzymes such as pyruvate dehydrogenase and α-KGDH are responsible for increasing NADH levels in the cell, favoring oxidative stress. Thus, one could suppose that thiamine deficiency also creates greater oxidative stress in the peritoneal cavity, therefore potentially inhibiting bacterial proliferation. The increased bacterial clearance that was observed in this study might initially suggest a better outcome against sepsis in these animals; however, the increased inflammation, as was observed with the increased TNF-α levels, may also be detrimental to sepsis outcomes. Indeed, it was reported that following CLP, an increased mortality rate occurred in animals that displayed an unbalanced inflammatory response, despite a greater bacterial clearance [53].

Extending the analysis of inflammation beyond the first 24 hours after the initiation of sepsis may present a more complete picture of the dynamics of the interleukins.

It is important to remember that the model of sepsis used in the present study can be adjusted to induce a low mortality rate, which may also allow for the investigation of thiamine deficiency in the mechanisms of immunologic tolerance.

## Conclusions

TD was associated with a greater bacterial clearance in the peritoneal fluid, greater oxidative stress and a change in the immune response profile in an experimental model of abdominal sepsis in mice. Further investigation is needed to define the impact of these changes on the severity of sepsis, septic mortality, and also to elucidate the participation of other cellular and biochemical mechanisms.

## Abbreviations

4-HNE: 4-hydroxy 2-nonenal; AIN93G: Kind of animal feed suggested by the American Institute of Nutrition in 1993; CFU: Colony-forming unit; CLP: Cecal ligation and puncture; GSH: Glutathione; HPLC: High performance liquid chromatography; IL-10: Interleukin-10; IL-1beta: Interleukin 1 beta; IL-6: Interleukin 6; iNOS: Inducible form of nitric oxide synthase; KC: Keratinocyte chemoattractant; MCP-1/CCL2: Monocyte chemotactic protein-1; NADPH: Nicotinamide adenine dinucleotide phosphate; NK: Natural killer cells; nmol/L: Nanomole per liter; ROS: Reactive oxygen species; SIRS: Systemic inflammatory response syndrome; TCA: Trichloroacetic acid; TD: Thiamine deficiency; TNF-alpha: Tumor necrosis factor alpha; TPP: Thiamine pyrophosphate; alpha-KGDH: Alpha-ketoglutarate dehydrogenase.

## Competing interests

The authors declare that they have no competing interest.

## Authors’ contributions

Conceived and designed the experiments: JAAA, VLFCB, JCBN, PTB and SC. Performed the experiments: JAAA, CRMG, NPAN, SCAJ, MGATC, RMC, and RNG. Analyzed the data: JAAA, MCP, VLFCB, JCBN, MBAML, CRMG, NPAN, SCAJ, MGATC, RM, RNG, PTB and SC. Contributed reagents/materials/analysis tools: MCP, VLFCB, JCBN, RNG, PTB and CAML. Wrote the paper: JAAA, CAML, PTB, and SC. All the authors have read and approved the final manuscript.
